# Topics of the Time

**Published:** 1898-02

**Authors:** 


### TOPICS OF THE TIME.

The christian-science editor of the *Buffalo Enquirer* has let himself loose on the bill proposed by the board of state medical examiners in Massachusetts, whereby it is sought to obtain a definition of what shall constitute the practice of medicine in that state. The following, according to the *Enquirer*, is the substance of the definition sought to be enacted into law:

> Any person shall be regarded as practising medicine within the meaning of this act who shall append to his name the letters M. D., or shall assume or advertise the title Dr. or physician, or any other title which shall show, or tend to show, that the person assuming or advertising the same is a practitioner of medicine, or of any of the branches of medicine, or who shall investigate or diagnose, or offer to investigate or diagnose, any physical or mental ailment or defect of any person with a view to affording relief, as commonly done by a physician or surgeon, or who shall prescribe for or treat a person for the purpose of curing any real or supposed disease, whether by the use of drugs or by the application of any other agency or alleged method of cure or alleviation or prevention of disease, or to operate as a surgeon for the cure or relief of any wound, fracture or bodily injury or deformity, after having received therefor, or with the intent of receiving therefor, either directly or indirectly, any bonus, gift or compensation.

The *Enquirer's* enthusiastic disciple of christian science thus comments on the Massachusetts' idea:

> Perhaps, if the christian scientists themselves established a standard of their own, thus preventing the gross charlatanism that has often been associated with it, as with nearly every radically new movement, the desired end would be reached. Christian science in the hands of an expert medical man can probably result in no harm. But the movement has the most to fear, not from antagonistic outsiders, but from quacks, who have allied themselves with the movement and under the protecting skirts of an earnest faith practise their incapacity with frightful results.

Many of the professional christian scientists have a legal M. D. which they append to their names ; many deprecate the use of a title. In Boston, which is one of the places where christian science finds very fruitful ground, considerable discussion has been stirred up by this suspected legislation.

It is undoubtedly advisable to have some standard of medical efficacy recognised by the profession whereby to separate the quack from the genuine. But if a discrimination is made against those who practise medicine in a fashion construed to be unorthodox by one body of doctors, trouble is bound to ensue.

It will be news to a large proportion of the intelligent world that so-called christian science is a "profession" in which there are "regulars" and "irregulars," scientists and quacks, M. D.'s and mountebanks ; but then the world moves. Perhaps the c. s. editor aforementioned may also learn something that is new to him by reading in our editorial pages the Kentucky definition of the practice of medicine.

---

THE law governing the practice of medicine in this state is being rigidly enforced through the efforts of the Medical Society of the County of New York. The society is splendidly organised and employs an able attorney at a reasonable and definite salary. Since the passage of the law in its present form a large number of arrests have been made and convictions obtained, while a number of cases are still pending. Fines of from $100 to $500 have been imposed upon druggists, pseudo-physicians, illegal practitioners and every other genus detected violating the law. One of the late cases was that of a druggist who was fined $150 for the illegal practice of medicine. This should be a warning to counter prescribing so frequently indulged in by druggists, but who in every instance become liable under the law.

Every county medical society in the state should instruct its board of censors to keep vigilant watch for violations of the practice act and to report all such to the proper prosecuting officer. With the advanced standards in medical education and the rigid guard placed at the entrance doors, a careful expurgation of all the illegal doctors should be made. The profession of medicine will then become honorable in the highest sense of the term.

